# Effects of a Mindfulness Intervention Among Arab Teachers Are Mediated by Decentering: A Pilot Study

**DOI:** 10.3389/fpsyg.2020.542986

**Published:** 2020-09-29

**Authors:** Aviva Berkovich-Ohana, Shiri Lavy, Kholoud Shanboor

**Affiliations:** ^1^Department of Learning, Instruction and Teacher Education, Faculty of Education, University of Haifa, Haifa, Israel; ^2^Department of Counseling and Human Development, Faculty of Education, University of Haifa, Haifa, Israel; ^3^Edmond Safra Brain Research Center, University of Haifa, Haifa, Israel; ^4^The Integrated Brain and Behavior Research Center (IBBRC), University of Haifa, Haifa, Israel; ^5^Department of Leadership and Policy in Education, Faculty of Education, University of Haifa, Haifa, Israel

**Keywords:** mindfulness, teachers, decentering, emotion regulation, arab schools

## Abstract

Although mindfulness-based interventions (MBIs) in education are widely spreading in the world, examination of mindfulness effects in Arab schools is still scarce. This pilot study aimed to fill this gap by examining the effects of an MBI among Arab teachers in Israel. This examination was conducted within the framework of the mindful self in school relationships (MSSR) model, which suggests that the positive effects of MBI on teachers’ emotion regulation are mediated by decentering. The participants (*N* = 39) were teachers from two Arab elementary schools in Israel, who underwent an MBI course (the MBI condition, *N* = 20) and another cognitive intervention (the control condition, *N* = 19). In a pre–post design, participants completed mindfulness, decentering, emotion regulation, and stress questionnaires. We hypothesized that (1) only in the MBI group, teachers’ mindfulness, decentering, and emotional regulation will increase and stress will decrease, and (2) changes in teachers’ decentering would mediate the associations of changes in teachers’ mindfulness with changes in their emotion regulation. ANOVA analyses show that, only in the MBI condition, teachers showed an increase in three mindfulness subscales (acting with awareness, non-reactivity, and observance), in decentering, and in adaptive emotion regulation (reappraisal) and a decrease in stress. Furthermore, changes from pre-intervention to post-intervention in teachers’ decentering mediated the associations of their pre–post changes in mindfulness with changes in emotion regulation. This study provides initial support to the feasibility and efficacy of MBI among Israeli Arab teachers and suggests decentering as a potential mediator of its effects in initial support of the MSSR model.

## Introduction

Mindfulness is a meditative practice originating in the Theravadan Buddhist tradition, defined as “paying attention in a particular way: on purpose, in the present moment, and non-judgmentally” (Kabat-Zinn, 2011, p. 291). In the last three decades, there has been a rapid growth of interest in mindfulness in educational settings (Meiklejohn et al., 2012; Roeser and Pinela, 2014) due to growing evidence for the contribution of mindfulness-based practices to attention and emotion regulation abilities and to increased physical and mental health (Roeser, 2014; Tang et al., 2015; Schonert-Reichl and Roeser, 2016). These compelling effects have motivated mindfulness cultivation in education and specifically among teachers [e.g., the Inner Resilience Program, Lantieri et al., 2016; the Cultivating Awareness and Resilience in Education (CARE), Jennings et al., 2013]. These mindfulness-based interventions (MBIs) for teachers’ professional development aim to reduce stress and improve performance and classroom environments by cultivating teachers’ mindfulness and emotion regulation. Indeed, research on MBIs for teachers shows that it enhances teachers’ well-being, performance (Hwang et al., 2017), teaching efficacy (Harris et al., 2016), and classroom management and promotes supportive relationships with students (Jennings, 2016) while reducing teacher burnout (Roeser et al., 2013).

Although MBIs in educational settings are becoming widespread in the world, especially in the United States and Europe (Ostafin et al., 2015) and, to a lesser extent, in Israel (Tarrasch, 2015; Ergas et al., 2018; Tarrasch et al., 2020), studies of mindfulness effects in Arab schools are still scarce. Although a few recent studies show a beneficial effect of MBIs on students in the United Arab Emirates (Nabulsi, 2015) and Saudi Arabia (Al-Ghalib and Salim, 2018), there is no report on such training targeting Arab teachers to the best of our knowledge. Furthermore, the vast majority of studies on teachers’ mindfulness are conducted in the United States and the United Kingdom (see Hwang et al., 2017). We found only one study that reported teachers’ MBI outcomes in an Islamic (but not Arab) country. In this Persian study (Jenaabadi et al., 2017), the 15 participants in the experimental group (but not the 15 in the control group) received eight sessions of training mindfulness skills. The results show a decrease in teachers’ stress and increase in teachers’ well-being in the MBI group compared to the control group. This scarcity of information about teacher MBI in diverse cultural contexts demonstrates the need for further examination of its outcomes and the underlying mechanisms in an educational context.

This is important, as cultural contexts differ in manners that are related to mindfulness. Specifically, Arab cultures differ from most Western cultures in a few ways that may impact the effect of mindfulness: (1) Arab cultures are more collective (Buda and Elsayed-Elkhouly, 1998) and interdependent within their in-groups (Triandis, 2001). In collectivist cultures (opposed to more individualistic cultures), people tend to prioritize the goals of their in-groups (e.g., family, tribe, nation) over their personal goals and are especially concerned about relationships (Triandis, 2001). (2) Arab cultures are typically more hierarchical than most Western cultures, and thus, not all people are considered equal (in essence), and the social structure is more strict and less flexible (Hofstede and McCrae, 2004). (3) There is higher uncertainty avoidance in Arab cultures which also typically means lower openness to changes and flexibility (Hofstede and McCrae, 2004). Such cultural differences are shown to affect social functions (e.g., Lavy et al., 2009) and propose different perceptions of the self, its importance, and its positioning in the social context (Shoshana, 2016). Furthermore, it can also be related to differences in well-being antecedents (Diener and Suh, 2003; Shoshana and Schade, 2018). Thus, the salutary effect of MBIs for teachers in Western society cannot automatically be generalized to Arab teachers and warrants research. The present pilot study aims to fill this gap by examining the effects of MBI in an Arab school.

The research was conceptualized within the novel framework of the mindful self in school relationships (MSSR) model (Lavy and Berkovich-Ohana, 2020). Briefly, the MSSR model delineates how the positive effects of teachers’ mindfulness in schools stem from the capacity of mindfulness to enhance teachers’ decentering, which, in turn, contributes to their relationship-promoting capacities because it increases their emotion regulation, empathy, and compassion abilities in interactions with students. Improved teacher–student relationships, in turn, contribute to teachers’ and students’ well-being and achievement (see Lavy and Bocker, 2018). This recent model (Lavy and Berkovich-Ohana, 2020) is theoretical, albeit solidly built on accumulating literature showing that mindfulness training shifts one’s self-awareness mode and the core understanding that education is an interpersonal, relational endeavor, which requires teachers to shift from a “self-centered” (or “self-focused”) processing mode in order to enable effective, nourishing teacher–student relationships (see also Berkovich-Ohana et al., 2019). Here, we assess only the first part of the model: examining the effects of MBI on mindfulness, decentering, emotion regulation, and stress and the role of decentering in mediating the effects of increased mindfulness on teachers’ emotion regulation and stress.

Decentering is defined as “the capacity to shift experiential perspective – from within one’s subjective experience onto that experience” (Bernstein et al., 2015, p. 599), i.e., shifting from identification with to observing of the experience or “the ability to step outside of one’s immediate experience, thereby changing the very nature of that experience” (Safran and Segal, 1996, p. 117). Decentering, considered a core underlying mechanism of mindfulness (Dahl et al., 2015), involves taking a non-judgmental attitude toward one’s inner experiences (Fresco et al., 2007). It involves a meta-cognitive awareness that one’s thoughts and emotions are only mental events, which can be observed without inner reaction (Shapiro et al., 2006; Grabovac et al., 2011; Bernstein et al., 2015). Decentering is shown to mediate the effects of MBIs (Carmody et al., 2009; Erisman and Roemer, 2010; Feldman et al., 2010) due to its explicit therapeutic effects, such as enhancing positive emotions, and reducing levels of distress, depression, and dysfunctional attitude (Hayes et al., 1999; Ashcraft and Moore, 2009; Salmon et al., 2009, Salmon et al., 2017; Hoge et al., 2015). In the present study, we suggest that decentering may also underlie the potential positive effects of MBI on emotion regulation.

Emotion regulation is generally defined as the processes by which individuals influence their emotions, emotional experiences, and emotional expressions (Gross, 1998). Emotion regulation is highly important for teachers because it enables them to relate to their experiences in a present-centered and responsive manner (Berkovich-Ohana et al., 2019) rather than in reactive ways that are past or future-oriented (Meiklejohn et al., 2012). The two key emotion regulation strategies most studied are expressive suppression and cognitive reappraisal (Gross and John, 2003). Cognitive reappraisal is the reinterpretation of a situation to alter its meaning and reduce its emotional impact, and expressive suppression is the attempt to inhibit, reduce, or hide emotion-expressive behavior; it is generally shown to have controversial effectiveness and negative health-related outcomes (Gross and John, 2003; Grandey and Melloy, 2017). Research generally shows that cognitive reappraisal is associated with more effective emotion regulation processes with fewer negative effects on the person conducting the regulation although both strategies can have psychological costs (Hülsheger and Schewe, 2011; Chang, 2013; Lavy and Eshet, 2018). Previous research has shown that MBI generally enhances teachers’ emotion regulation skills (Meiklejohn et al., 2012; Flook et al., 2013).

The specific aim of this pilot study was to explore the effectiveness of MBI among Arab teachers in Israel. Specifically, we hypothesized that (1) the MBI would result in enhanced levels of mindfulness, decentering, and emotion regulation and reduced stress, and (2) building on the MSSR model, the positive effects of enhanced mindfulness on emotion regulation would be mediated by teachers’ decentering.

## Materials and Methods

### Study Procedure and Interventions

In Israel, all schools and teachers must periodically undergo continuing education programs (in various topics), which typically include 30 training hours and are given either by private organizations or through the Development Education Personnel (PISGA). These programs are often given to all schoolteachers in the school. For this study, two Arab schools were chosen (and compared) due to their similarities: Both were elementary schools (grades 1–8), serving 300 (MBI school) or 242 (control school) students mostly with low socioeconomic status (lowest 30% according to the Ministry of Education index).

The MBI was a 30 h, 3-month training course, called Applied Mindful Pedagogy for Educators and aimed at developing mindfulness and reducing stress. Its main components were (a) group activities, including experiential practices, group discussions, lectures on stress and forgiveness, etc.; (b) mindfulness practices, including, for example, attentional focus on the breath, body scan, and monitoring experience with the aim to develop concentration and non-reactivity; and (c) homework assignments, including daily formal mindfulness practices, journaling, and other weekly assignments (e.g., loving kindness for particular students) (for more details, see Supplementary Appendix). The control intervention was a 30 h, 6-month training course given by Branco Weiss^1^ called Teaching for Understanding, aimed at promoting constructivist teaching and in search of understanding and development of higher order thinking. All participants completed questionnaires at three time points: a week before the interventions started (Time 1, T1), after 3 months at the end of the MBI intervention (Time 2, T2), and after 6 months at the end of the control intervention (Time 3, T3). The study was approved by the University of Haifa IRB committee.

### Participants

All teachers involved in this study comprised a convenience sampling, most suitable for pilot studies (Etikan et al., 2016). None of the participating teachers had any prior mindfulness training. The mindfulness intervention (MI) group comprised 20 teachers, out of which 12 completed the intervention. The control (C) group comprised 19 teachers, all of which completed the intervention (see Supplementary Table 1 for complete demographic details and study limitations for explanation of the compliance discrepancy). All participants signed a consent form.

### Research Tools

We used Hebrew forms of the questionnaires, which were validated in previous studies, as Arab teachers in the mixed cities (in which the study was conducted) are fluent in Hebrew due to daily contact with Hebrew-speaking neighbors (Mar’i, 2013). Apart from a demographic questionnaire, we used the following scales (reliability scores in our sample are given in Table 1):

1. Perceived Stress Scale (PSS, reliability 0.85; Cohen et al., 1983), in which participants rank on a scale ranging from 1 = almost never to 5 = very often the extent to which they feel in the ways described in each of the 14 items (e.g., “nervous and stressed”).
2. Experiences Questionnaire (EQ, reliability 0.81–0.84; Fresco et al., 2007), designed to measure decentering with two subscales: decentering – the realization that thoughts, feelings, and reactions are transitory patterns of mental activity and that they are not necessarily true representations of the self and events (e.g., “I notice that I don’t take difficulties so personally”) – and rumination, which reflects disengagement from habitual ruminative thoughts (e.g., “I think over and over again about what others have said to me”). The measure comprises 19 items, which are ranked on a scale ranging from 1 (never) to 7 (always).
3. The Emotion Regulation Questionnaire (ERQ, Gross and John, 2003), a 10-item measure assessing two emotion regulation dimensions: cognitive reappraisal (reliability 0.79, e.g., “When I want to feel less negative emotion (such as sadness or anger), I change what I’m thinking about”) and expressive suppression (reliability 0.73, e.g., “I control my emotions by not expressing them”). Participants ranked their agreement with each statement on a scale ranging from 1 = strongly disagree to 7 = strongly agree.
4. The Five Facet Mindfulness Questionnaire (FFMQ, reliability range for all facets 0.72–0.92; Bohlmeijer et al., 2011), which is a 24-item questionnaire that assesses five mindfulness facets: observing (e.g., “I pay attention to physical experiences, such as the wind in my hair or sun on my face”), describing (e.g., “I’m good at finding words to describe my feelings”), acting with awareness (e.g., “I find it difficult to stay focused on what is happening in the present moment” – reversed item), non-judging of inner experience (e.g., “I tell myself I shouldn’t be feeling the way I’m feeling” – reversed item), and non-reactivity to inner experience (e.g., “When I have distressing thoughts or images, I don’t let myself be carried away by them”). Participants rate the degree to which each statement is true for them on a scale ranging from 1 = never or very rarely true to 5 = very often or always true.

**TABLE 1:** Descriptive statistics and differences within and between groups for all questionnaires’ subscales.

Questionnaire	Measure	Alpha cronbach	Group P	Time 1 (*M ± SD*)	Time 3 (*M ± SD*)	Paired-samples *t*-test Time/Group (*t*-values)	Independent samples *t*-test Time1 (*t*-values)	Independent samples *t*-test Time 3 (*t*-values)
Five Facets	Total	0.69	MI	76.50 ± 6.03	85.41 ± 3.96	4.29**	2.06*	3.93***
Mindfulness			C	82.78 ± 9.39	74.00 ± 5.72	7.12***		
Questionnaire	Act-aware	0.86	MI	17.83 ± 3.56	20.50 ± 1.38	2.49*	.03	4.95***
			C	17.78 ± 4.10	15.31 ± 3.43	3.94**		
	Non-react	0.57	MI	14.00 ± 1.47	16.33 ± 2.49	3.38**	1.48	2.86**
			C	15.47 ± 3.22	14.05 ± 1.92	3.04**		
	Non-judge	0.51	MI	16.5 ± 2.67	16.5 ± 3.11	0.00	1.27	2.07*
			C	15.26 ± 2.76	14.52 ± 2.19	2.16*		
	Describe	0.54	MI	15.25 ± 3.33	17.50 ± 2.50	2.13	3.56**	0.89
			C	18.84 ± 2.29	16.73 ± 2.20	6.33***		
	Observe	0.83	MI	11.50 ± 4.25	14.58 ± 2.19	2.55*	2.89**	1.28
			C	15.42 ± 3.27	13.36 ± 2.75	4.02**		
Experience	Decentering	0.80	MI	50.08 ± 7.12	78.41 ± 9.30	−8.86***	0.07	−9.79***
Questionnaire			C	17.78 ± 4.10	44.26 ± 9.23	0.83		
scores	Rumination	0.65	MI	42.66 ± 5.98	19.75 ± 4.30	9.15***	0.66	8.35***
			C	43.84 ± 7.88	37.15 ± 6.33	2.60*		
Emotion-	Cognitive	0.66	MI	5.22 ± 0.72	5.70 ± 0.36	2.42*	0.69	−6.77***
Regulation	Reappraisal		C	5.41 ± 0.75	4.38 ± 0.60	12.53***		
scores	Expressive	0.69	MI	4.12 ± 0.65	4.08 ± 0.62	0.18	0.62	−1.29
	Suppression		C	4.36 ± 1.23	3.72 ± 0.82	5.52***		
Stress Scores	Total	0.83	MI	26.50 ± 1.53	17.16 ± 1.39	3.87**	2.92**	2.23*
			C	20.50 ± 1.42	23.15 ± 1.48	−1.255		

**Mindfulness intervention group (MI, n = 12), and Control group (C, n = 19). ^∗^p < 0.05; ^∗∗^p < 0.01; ^∗∗∗^p < 0.001.**

### Statistical Analyses

Before examining the intervention effects, we first compared scores from T2 and T3 in both groups. We found no significant differences between T2 and T3 in any of the dependent measures within each group, suggesting that no significant differences occurred between the end of the MBI and the end of the control intervention. Thus, we report only the scores from T1 and T3 (pre–post).

We examined the effects of MBI by conducting a set of repeated-measures ANOVAs for group (MI, C) by time (T1, T3), separately for each questionnaire, supplemented with *post hoc* independent samples *t*-tests for group comparisons and paired *t-*tests for time comparisons. In order to examine the mediation hypothesis for the changes from pre to post measurement (T3 minus T1), a parallel multiple mediation model was estimated for all study participants (*N* = 31), using ordinary least squares path analysis to determine the effect of change in mindfulness levels on change in emotion regulation through changes in decentering and changes in rumination. These two models were examined using PROCESS (Hayes, 2012; Model 4) to estimate the indirect effects of each mediator; 5,000 bias-corrected bootstrap samples were used for the 95% confidence interval (CI).

## Results

Preliminary analyses indicate no differences between the groups in most demographic parameters (years of education, teaching hours per week, gender, religion). However, there were significant differences in age and in teaching experience: MI participants were younger and had less teaching experience than the control group participants (Table 1).

### Changes in Mindfulness Scores (FFMQ)

**FIGURE 1:**
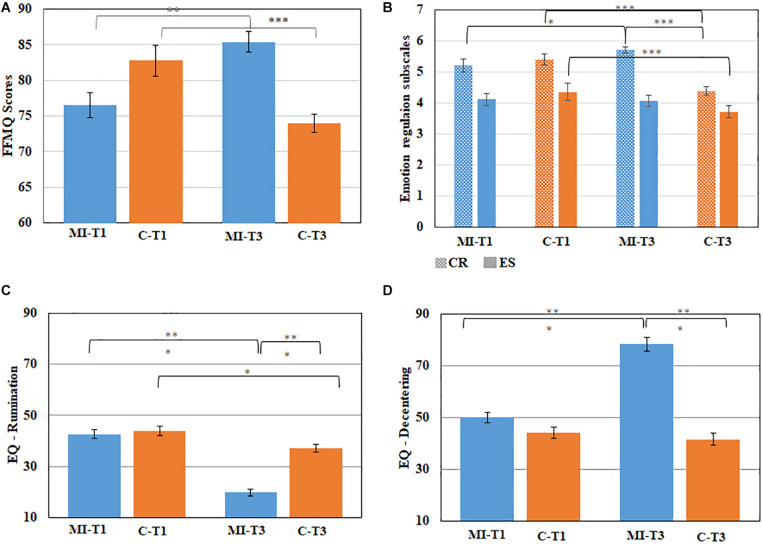
Questionnaire scores. Significant interactions for Group [the mindfulness intervention group (MI, *n* = 12), and the Control group (C, *n* = 19)] and Time [Time 1 (T1, pre-intervention), and Time 3 (T3, post-intervention)], for the following scales (*M ± SEM*): **(A)** Five Facets Mindfulness Questionnaire (FFMQ); **(B)** Emotion Regulation Subscales. CR, Cognitive Reappraisal; ES, Expressive Suppression; **(C)** Rumination scores; **(D)** decentering scores. Blue and orange denote the MI and control groups, respectively, *p < 0.05; **p < 0.01; ***p < 0.001.

A three-way, repeated-measures ANOVA was conducted with one grouping variable, Time, and FFMQ subscales (Observe, Describe, Act with Awareness, Non-judgement, Non-reactivity). A main effect was uncovered for the FFMQ subscales (lowest scores for Observe, highest scores for Describe and Act Aware). In addition, we found a significant Group × FFMQ subscales interaction [*F*(4, 116) = 3.80; *MSE* = 19.83; *p* < 0.05], stemming from generally higher scores for Act Aware, Non-judge, and Non-react in the MI vs. C group and the opposite for Observe and Describe. More importantly, we found a significant Group × Time interaction [*F*(1, 29) = 67.48; *MSE* = 3.89; *p* < 0.001], where FFMQ scores generally increased from T1 to T3 for the MI group and vice versa for the C group (Figure 1A and Table 1). Indeed, *post hoc t*-tests show a significant increase in overall mindfulness for the MI group [*t*(11) = 4.29; *p* < 0.01] as well as a significant decrease in the C group [*t*(18) = 7.12; *p* < 0.001]. The detailed effect of MBI can be best seen in the significant Group × Time × FFMQ subscales interaction [*F*(4, 116) = 3.69; *MSE* = 3.64; *p* < 0.01]. *Post hoc t*-tests for this interaction show that all 5 FFMQ facets significantly decreased in the C group, and 3 facets (Act with Awareness, Non-reactivity, and Observe) significantly increased in the MI group. It should be noted that there was a significant difference between the groups for T1 for 2 FFMQ facets (Describe and Observe) with the MI group scoring lower than the C group; thus, this did not drive the effect of enhanced mindfulness in the MI group following the intervention but rather supports and emphasizes it. As for T3, there was a significant difference between the groups in the 3 other FFMQ facets (Act with Awareness, Non-judge, Non-react), not observed for Describe and Observe (perhaps due to the opposite difference between them in T1).

### Changes in Emotion Regulation Scores

A three-way ANOVA was conducted with one grouping variable (MI, C), Emotion Regulation subscales (Cognitive Reappraisal, Expressive Suppression), and Time (T1 and T3). The results indicate a main effect [*F*(2, 58) = 33.30; *MSE* = 1.08; *p* < 0.001] as Expressive Suppression scores were generally lower than Cognitive Reappraisal scores, and a significant main effect for Time [*F*(1, 29) = 17.91; *MSE* = 0.15; *p* < 0.001] as generally scores were lower in T3. We also found a Group **×** Time interaction [*F*(1, 29) = 53.28; *MSE* = 0.15; *p* < 0.001]; Emotion Regulation scores generally increased from T1 to T3 for the MI group but decreased for the C group. Aligned with our hypotheses, we found a significant Group **×** Time **×** Emotion Regulation interaction [*F*(2, 58) = 8.57; *MSE* = 0.17; *p* < 0.01] (Figure 1B and Table 1). *Post hoc t*-tests show a significant increase in Cognitive Reappraisal scores [*t*(11) = −2.4; *p* < 0.05] from T1 to T3 in the MI group although, for the C group, there was a decrease in both Cognitive Reappraisal [*t*(18) = 12.53; *p* < 0.001], and Expressive Suppression scores [*t*(18) = 5.52; *p* < 0.001]. At T3, there was a significant difference between the groups only for Cognitive Reappraisal scores [*t*(29) = 6.77; *p* < 0.001].

### Changes in Decentering and Rumination (EQ) Scores

A three-way ANOVA was conducted with one grouping variable, Time, and repeated measures on EQ scores (Rumination and Decentering). Results indicate a main effect for the EQ subscales as Rumination scores were generally lower than Decentering. We also uncovered a main effect for Group [*F*(1, 29) = 13.76; *MSE* = 77.05; *p* < 0.01] as the EQ scores were generally higher for the MI group. In addition, we found a significant Group × EQ subscales interaction [*F*(1, 29) = 143.81; *MSE* = 47.86; *p* < 0.001], stemming from higher scores for Decentering in the MI group and the opposite for Rumination. We also found a Group **×** Time interaction [*F*(1, 29) = 4.44; *MSE* = 89.71; *p* < 0.05], where EQ scores generally changed from T1 to T3 for the MI group but did not change for the C group.

Importantly, in support of our hypotheses, we found a significant Group **×** Time **×** EQ subscales interaction [*F*(1, 29) = 90.17; *MSE* = 45.42; *p* < 0.001]: for the MI group, there was a significant increase in Decentering [*t*(11) = 8.86; *p* < 0.001] from T1 to T3 and a significant decrease in Rumination [*t*(11) = −9.1; *p* < 0.05], and for the C group, there was only a significant decrease in Rumination [*t*(18) = 2.60; *p* < 0.05] and no change in Decentering (Figures 1C,D and Table 1). It should also be noted that there was a significant difference between the Groups at T3 for both Decentering [*t*(29) = 9.79; *p* < 0.001] and Rumination [*t*(29) = 8.35; *p* < 0.001] in support of our hypotheses.

### Changes in Stress Scores

A three-way ANOVA was conducted with one grouping variable, Time and Stress Scores. We found a significant Group **×** Time interaction [*F*(1, 29) = 11.93, *MSE* = 46.87; *p* < 0.01], indicating that Stress scores changed from T1 to T3. *Post hoc t*-tests suggest that this change was driven by a significant decrease in stress at the MI group between T1 and T3 [*t*(29) = 3.87; *p* < 0.01] (Table 1). There was also a significant difference between the groups at T1 [*t*(29) = 2.92; *p* < 0.01] and at T3 [*t*(29) = 2.23; *p* < 0.05] (Table 1).

### Mediation Results

**FIGURE 2:**
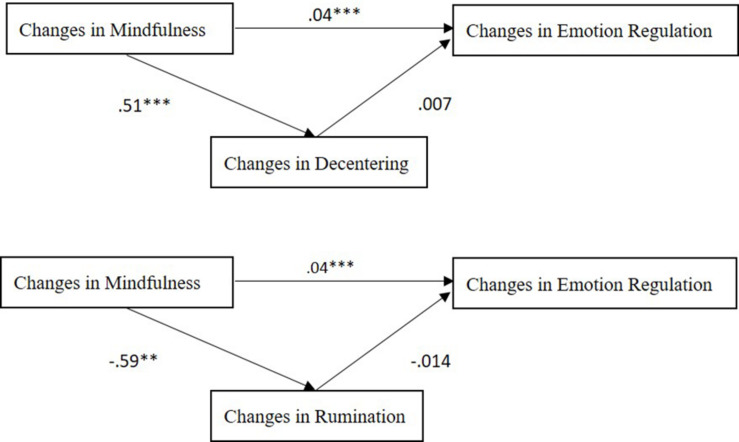
Mediation analyses. Results of the mediation analyses (*n* = 31) examining the role of changes in decentering and in rumination in mediating the association of changes in mindfulness with changes in emotion regulation (from T1 to T3); **p < 0.01; ***p < 0.001.

The unstandardized correlations and bootstrap solutions of the analyses are presented in Table 2 (see also Figure 2). Only one model supported the mediation hypothesis (i.e., the first model), indicating a significant path from changes in mindfulness (from T1 to T3) to changes in emotion regulation and mediation of this path via changes in decentering (Table 2; zero was not within the 95% confidence intervals in all three analyses).

**TABLE 2:** Mediation model for predicting changes in teachers’ emotion regulation from changes in teachers’ mindfulness with the changes in decentering and rumination as mediators.

Mediation model with decentering as mediator			

	Bootstrap 95% CIs (LLCI, ULCI)	Effect	SE
Direct effect of mindfulness		0.04***	0.01
Indirect effect of mindfulness via decentering	[0.0002, 0.019]	0.008	0.005
**Mediation model with rumination as mediator**			
Direct effect of mindfulness		0.04***	0.01
Indirect effect of mindfulness via rumination	[−0.0003, 0.022]	0.008	0.006

*95% confidence intervals are presented in brackets; LLCI, lower-level confidence interval; ULCI, upper-level confidence interval; Confidence intervals that do not include 0 (null association) are significant; Coefficients for all models are presented in Figure 2.* ***p < 0.01.

## Discussion

This pilot study examined the effects of MBI among Arab teachers in Israel and explored the role of changes in decentering in mediating the associations between changes in teachers’ mindfulness and changes in their emotion regulation. As such, the study provides an initial understanding of potential teachers’ MBI effects in this cultural context. Teachers in Israel generally face challenges that differ from those in other countries (e.g., the United States and the United Kingdom) in which teacher MBI is usually explored; see review by Tarrasch et al. (2020). Such challenges include, for example, larger classes, more violence, and lower payment compared to the average in the Organization for Economic Co-operation and Development (OECD, 2011, 2019). Within Israel, Arab schools also suffer from decreased budgets and increased pedagogical difficulties (Viniger, 2018). Such contextual factors may lead to differences in teacher’s attitudes as supported by a few previous studies showing that Israeli teachers exhibit stricter classroom discipline strategies compared to other teachers (e.g., Australian teachers; Lewis et al., 2005). Arab teachers may also tend to use more hierarchical management strategies, typical in hierarchical collectivistic cultures. In the present study, we focus on the effects of an MBI among Arab teachers as they have not been explored before despite the large Arab population (without comparing them directly to other populations).

As hypothesized, we found a significant increase following MBI in teachers’ mindfulness scores in three mindfulness subscales (Acting with Awareness, Non-reactivity, and Observe), and there was a significant decrease in all mindfulness subscales in the control group. These findings are partially consistent with the results of previous studies (Bohlmeijer et al., 2011) showing that these three mindfulness facets (Acting with Awareness, Non-judging, and Non-reactivity) often change as a result of MBI, and the other two facets (Observing and Describing) are less susceptible to change. However, others (Carmody et al., 2009) report moderate-to-large effect sizes in all facets following an MBI. Failing to find effect in all facets of FFMQ in our study might be due to the small sample size. In support of this explanation, the Describe scale did show a strong trend, which did not reach significance. Finally, our results are strongly aligned with a recent study in Israel testing the effect of the “Call to Care – Israel for Teachers” (C2CIT) program, which employs mindfulness, compassion, and social – emotional skill training (Tarrasch et al., 2020). In a slightly larger group size (starting with 20, but ending with 17 in the intervention group and starting with 24 and ending with 22 in the control group), the authors found a significant increase following MBI among Jewish teachers’ mindfulness scores in three mindfulness subscales – the first two identical to our findings (Acting with Awareness, Non-reactivity, and Describe). Future studies with larger samples may shed light on this issue.

We observed a significant change in EQ scores following the intervention only in the MI group, including an increase in Decentering and a decrease in Rumination as hypothesized. These findings correspond with previous studies (Shapiro et al., 2006; Fresco et al., 2007) that demonstrated MBIs cultivate a fundamental shift in decentering. The fact that we found a significant effect in such a small group size demonstrates the strong effect that MBI may have on enhancing decentering and reducing rumination.

We hypothesized, based on previous literature reviews (Chambers et al., 2009), that MBI would promote emotion regulation in the MI group. The findings indicate that the MBI contributed only to increased use of Cognitive Reappraisal and not to changes in the use of Expressive Suppression. Failing to show reduced Expressive Suppression following MBI can stem from the small group size. At the same time, there was a significant decrease in both Expressive Suppression and Cognitive Reappraisal in the control group after the control intervention, suggesting that this intervention also facilitated emotional regulation and enabled reduced use of both strategies.

Interestingly, emotion regulation rankings in the study were significantly higher than reported in the literature for Western cultures. For example, Cognitive Reappraisal values were higher than 5.2 for both groups and, for the Expressive Suppression, higher than 4.1, and they were reported for Westerners as 4.6 and 3.36, respectively (Moore et al., 2008). A possible explanation can be due to cultural differences between Western and Arab cultures, the latter showing a strong norm for emotional moderation (Diener et al., 2003), possibly stemming from the need to maintain cooperation and harmony (Noon and Lewis, 1992; Mesquita and Delvaux, 2013). In Arab cultures, emotions are related to the social context and, thus, less related to the inner self (Safdar et al., 2009); hence, they tend to be more controlled.

As for the stress scores, although the MI group showed significantly enhanced stress scores pre-training compared to the control group, possibly related to their younger age and lower teaching experience, they showed significantly reduced stress post-intervention in contrast to the controls. This is aligned with another recent study on Israeli teachers (Tarrasch et al., 2020).

Finally, Decentering was found to mediate the relationship between mindfulness and Emotion Regulation in initial support of the first part of the MSSR model (Lavy and Berkovich-Ohana, 2020), suggesting that mindfulness effects on teachers’ social capacities are mediated by decentering. Indeed, this result is aligned with previous studies showing the contribution of decentering to emotion regulation, specifically linking it with reduced anxiety and depression (Hoge et al., 2015) and enhanced mental health (Hayes et al., 1999; Kabat-Zinn, 2009). The study suggests that further exploration of decentering and its role in fostering mindfulness’ positive outcomes may be worthwhile even in collectivist cultures.

To summarize, the results of our pilot study provide initial evidence that a 30 h MBI can enhance mindfulness, decentering, and emotion regulation as well as reduce stress among Arab teachers, indicating a potential positive effect of MBIs in this population (beyond the cultural differences). The relevance of the results to Arab teachers is very high, considering the novelty of MBIs in Arab societies and the scarcity of previous publications in this population. The current pilot study supports the results of previous studies demonstrating the benefits of mindfulness practice in reducing educators’ stress as well as enhancing their mindfulness and emotion regulation. Considering the larger classes, lower payment, and higher violence in Israel compared to other Western countries (OECD, 2011, 2019), these results are highly promising, considering both the short intervention (30 h), and the non-elective nature of the intervention for the teachers, which is, by large, the situation in many other schools. Finally, our results provide initial support to one part of the MSSR model, warranting further investigation.

### Study Limitations and Future Directions

The major limitation of our study is the small group size, which limits its generalization ability. However, considering the total lack of reports on MBI in Arab teachers, we believe that this pilot study points toward promising potential, albeit cultural differences. Another limitation is the nature of the groups; the interventions were given to the teachers within the school, comprising a convenience sampling. Hence, we can make only weak statements about the population of interest (Etikan et al., 2016). Yet convenience sampling in this case was affordable, and the subjects were readily available through the school manager’s cooperation. Importantly, the sample does not differ much from the population of interest as it is often the case that MBIs in schools are mandatory. Thus, this design can give a realistic view as often the case is a mandatory school intervention to which the teachers need to accommodate. A related limitation is the initial demographic difference between the groups as teachers in the MI group were younger and had less teaching experience (Table 1) and, not surprisingly, also showed less compliance to complete the training (compared to the control group). The reason is that young teachers in Israel are required to attend more continuing education training hours and simultaneously took such additional training both within and outside school. Thus, some teachers in the MBI group were obliged to miss some of the sessions (due to conflicts with their other training). They were excluded from the study after two absences. The older teachers in the C group needed less simultaneous continuing education, enabling all of them to complete the training.

## Data Availability Statement

The original contributions presented in the study are included in the article/Supplementary Material, further inquiries can be directed to the corresponding author/s.

## Ethics Statement

The studies involving human participants were reviewed and approved by the University of Haifa IRB committee. The patients/participants provided their written informed consent to participate in this study.

## Author Contributions

AB-O: conceptualization, supervision, writing – original draft. KS: data curation, formal analysis, investigation, writing – review. SL: writing – original draft, and formal analysis. All authors contributed to the article and approved the submitted version.

## Conflict of Interest

The authors declare that the research was conducted in the absence of any commercial or financial relationships that could be construed as a potential conflict of interest.

## Footnotes

1 https://brancoweiss.org.il/en/
