# Supplementary material for: Effects of a Mindfulness Intervention Among Arab Teachers Are Mediated by Decentering: A Pilot Study
Source: Front Psychol. 2020 Sep 29;11:542986. doi: 10.3389/fpsyg.2020.542986 (PMC7550638; doi:10.3389/fpsyg.2020.542986)
Supplement: Supplementary file 1 [file Data_Sheet_1.docx]

- 1. ***Appendix I: Mindfulness –based intervention program***

This program was a tailor-made by Dr. Galit Kliger for teachers in schools, given for the first time. Since then, it was given again in 2020 through PISGA. Importantly, this 30-hours basic course was based on a longer 120-hour (60 per year for 2 years) mindfulness-based intervention given yearly by Dr. Galit Kliger through PISGA since 2015.

This MBI, based upon Jon Kabat-Zinn’s widespread Mindfulness-Based Stress Reduction (MBSR) program, uses approximately 90 % of the same mindfulness meditation practices, emotion skills and theory and practice of compassion and forgiveness. About 5 % of the program is devoted to Adlerian psychology strategy to emotion theory regulation (marked by a in the table). The remaining 5 % of the program focuses on neuro-linguistic programming (marked by b in the table). An overview of the MBI sessions, curriculum, and depth of coverage is presented in Table1.

The main program components fall into three categories:

(a) Group activities: Group activities include visualizations, experiential exercises, small and large group discussions of practice, didactic lectures on topics like stress and forgiveness, and guided mindfulness and “heart” practices.

(b) Mindfulness practices: Mindfulness practices include specific mental training exercises like an attentional focus on the body, the breath, or the ongoing flow of experience that aim to develop concentration, clarity of perception, and non- reactivity.

(c) Homework assignments: Homework includes things like daily mindfulness practice, keeping a meditation journal, and engaging in weekly homework assignments (e.g., doing loving-kindness practice for a challenging student for one week and writing about the experience). Each teacher submitted 5 home assignments, each received a feedback from the instructor, and a final written reflection.

Table 1: A short description of the program’s curricular schedule for the 10 meetings.

| **Group activity** | **Mindfulness practice** |
| --- | --- |
| Welcome & overview  Factors influencing a pleasant learning environment, in relation to mindfulness  “Hot cross bun” - Our internal patterns (thoughts, reactions, sensations, actions) are trainable and amenable to change | Introduction to formal breath training |
| Neuroplasticity – the brain is amenable to training  What is mindfulness – definitions, cognitive scientific evidence | Distinguishing between formal and informal practice  Mindful eating – the raisin exercise  Awareness to breath |
| “Automatic pilot” – identifying internal patterns  Anchors for attention – breath and body sensations | Body scan |
| Adler’s behavioral model (event-interpretation-emotion-reaction) ^a^  Workshop – identifying our automatic patters | Awareness to breath with anchors (breathing like a wave) |
| Identifying the internal families of ‘guests’(craving, aversion, restlessness, boredom, doubt) | Movement practice |
| Meeting the guests with mindfulness  Introducing ‘RAIN’ model (recognize, accept, inquiry, non-identify) | Practicing ‘like a mountain’ |
| Stress, anxiety – their role in our lives, effective levels  Chronic vs. acute stress  The contribution of mindfulness to stress coping strategies | Breathing space in three steps |
| Workshop – reconnection to internal resources ^b^  Thankfulness and gratitude | Connecting to resources using metaphors  Gratitude practice |
| Fostering compassion and heartfelt qualities  The permission to be human  Empathy and sympathy | Loving-kindness practice (towards a loved and neutral person) |
| Summary – content and experience  Suchness  Reflection & Feedback  So what can we do with mindfulness in the classroom? | Open awareness |
